# Effects of exercise based on ACSM recommendations on individuals with a prediabetic state: a systematic review and meta-analysis of randomised controlled trials

**DOI:** 10.1530/EC-25-0270

**Published:** 2026-01-02

**Authors:** Feifei Huang, Yu Ling, Liying Chen

**Affiliations:** ^1^ Ultrasound Medicine Department, Zhejiang University School of Medicine Sir Run Run Shaw Hospital, Hangzhou, China; ^2^ General Practice Department, Zhejiang University School of Medicine Sir Run Run Shaw Hospital, Hangzhou, China

**Keywords:** ACSM recommendations, exercise, prediabetic state, meta-analysis, RCT

## Abstract

**Background:**

This systematic review seeks to employ a meta-analysis to evaluate the differential impacts of exercise interventions with high adherence versus those with low or uncertain adherence, following the American College of Sports Medicine (ACSM) guidelines, on factors such as glucose regulation and insulin sensitivity in prediabetic patients.

**Methods:**

A thorough search was executed across databases such as PubMed, Embase, Web of Science, and the Cochrane Library from inception to September 1, 2025. We carried out a meta-analysis concentrating on exercise interventions among prediabetic patients. Only randomized controlled trials were included in our study. The meta-analyses determined WMDs for parameters such as fasting blood glucose (FBG), 2-hour plasma glucose (2hPG), glycated haemoglobin A1c (HbA1c), and homoeostasis model assessment of insulin resistance (HOMA-IR).

**Results:**

There were 24 studies with a total of 1,480 participants. Compliance with the ACSM recommendations was categorised as ‘high’ in 9 studies and ‘low or uncertain’ in 15 studies. In the subgroup analysis comparing high compliance with ACSM recommendations to low or uncertain compliance, the results were as follows: FBG: −0.55 (95% CI: −1.01, −0.09) vs −0.42 (95% CI: −0.62, −0.21), 2hPG: −0.97 (95% CI: −1.48, −0.45) vs −0.70 (95% CI: −1.15, −0.25), HOMA-IR: −0.47 (95% CI: −0.81, −0.14) vs −0.50 (95% CI: −1.09, 0.08), HbA1c:−0.20 (95% CI: −0.50, 0.10) vs −0.29 (95% CI: −0.46, −0.12).

**Conclusion:**

Subgroup analyses were conducted to evaluate whether intervention adherence modifies the metabolic benefits of exercise. Mean reductions in FBG, 2hPG, and HOMA-IR were numerically larger in participants with high, compared with low or uncertain, adherence, suggesting a possible dose–response relationship between adherence and glycaemic improvement. Conversely, HbA1c displayed a marginally greater reduction in the low-adherence stratum – an observation that contrasts with the pattern seen for the other glucose/insulin indices. This discrepancy may reflect greater analytical variability of HbA1c, differences in intervention duration, baseline imbalance, or heterogeneity in programme content and individual responsivity across adherence categories. Large-scale, long-term trials that standardise the measurement of adherence and the delivery of exercise interventions are needed to definitively clarify the association between adherence and metabolic outcome.

## Introduction

The World Health Organization (WHO) defines prediabetes as a condition characterised by fasting blood glucose (FBG) levels between 6.1 and 7.0 mmol/L, 2‐hour plasma glucose (2hPG) levels between 7.8 and 11.1 mmol/L, and/or glycated haemoglobin A1c (HbA1c) levels between 6.0 and 6.4% (1). Individuals with prediabetes are at high risk of progressing to type 2 diabetes mellitus (T2DM) (2) and are also associated with cardiovascular diseases (CVDs) and increased all-cause mortality (3), imposing a significant health burden on society and families. Prediabetes can persist or resolve to a normal state. The increasing prevalence of prediabetes represents a significant global health concern, affecting a current population of 415 million with diabetes, projected to reach 642 million by 2040 (4). The literature suggests that weight loss, lifestyle interventions, and metformin therapy can reduce diabetes progression in individuals with impaired glucose tolerance (5, 6, 7, 8).

Exercise is a planned, organised, and repetitive physical activity aimed at improving or maintaining one or more dimensions of physical fitness (13, 14). Exercise therapy is widely utilised due to its ease of implementation and cost-effectiveness. Moreover, the wide variety of exercises allows individuals to select a suitable regimen based on their interests or physical condition. The US Preventive Services Task Force recommends exercise therapy as an effective treatment for patients with prediabetes and T2DM (9, 10). Existing research indicates that exercise interventions play a crucial role in reversing prediabetes. Exercise interventions such as resistance training (RT), aerobic training (AT), Yijinjing, Baduanjin, among others, can reduce FBG, 2hPG, homoeostasis model assessment of insulin resistance (HOMA-IR), and HbA1c (11, 12, 13, 14, 15, 16).

It is unclear whether differences among studies are due to exercise intensity, frequency, or duration. There is currently no established recommendation for the optimal exercise dosage in prediabetes. The American College of Sports Medicine (ACSM) has developed a comprehensive physical activity prescription for apparently healthy adults, which includes detailed recommendations for cardiorespiratory exercise, resistance exercise, and flexibility exercise (17), which are detailed in Table 1. A meta-analysis is a statistical method used to synthesise findings from multiple studies on the same topic, potentially resolving discrepancies among them (18). The purpose of this review is to conduct a meta-analysis comparing the effects of exercise interventions with high compliance versus low or uncertain compliance with ACSM recommendations on glucose metabolism and insulin resistance in individuals with prediabetes.

**Table 1:** The ACSM recommendations for cardiorespiratory fitness, muscular strength, and flexibility in apparently healthy adults.

Exercise dose	Cardiorespiratory exercise	Resistance exercise	Flexibility exercise
Frequency	4–5 days/week	1–2 days/week (non-consecutive days) gradually increasing to 2–3 days/week	5–7 days/week
Intensity/workload	Moderate intensity, 40–59% VO2R/HRR, Cr-10 scale rating of 3–4	Adjust the resistance, with the last two sets being challenging. High intensity training can be performed if tolerable	Stretch until you feel your muscles being pulled tight or a slight discomfort
Duration	Gradually increase from 20 min to at least 30 min (up to 45–60 min)	Starting with one set of 8–12 repetitions, increase to two sets after about 2 weeks. Perform no more than 8–10 exercises per session	Static stretching held for 10–30 s, repeated 2–4 times

HRR, heart rate reserve; VO2R, oxygen uptake reserve.

## Materials and methods

The protocol will adhere to the Preferred Reporting Items for Systematic Reviews and Meta-Analyses (PRISMA) guidelines and has been registered in Prospective Systematic Reviews (PROSPERO: CRD42024556803).

### Search strategy

PubMed, Embase, Web of Science, and the Cochrane Library were searched from inception to September 1, 2025, following the PICOS principle with a focus on study population, interventions, and research methodology. The detailed search strategy is outlined in Supplementary Appendix 1 (see section on Supplementary materials given at the end of the article). Furthermore, we employed a snowballing technique: the reference lists of all included studies were manually screened to identify additional eligible reports, with particular attention to the grey literature, and corresponding authors were contacted when supplementary unpublished or hard-to-retrieve data were suspected to exist.

### Criteria for selection of studies

The included studies had to meet the PICOS criteria. Specifically: i) individuals who have prediabetes; ii) the intervention period of exercise should be no less than 1 month, and the types of exercise covered aerobic exercise, resistance exercise, and stretching exercise; iii) control groups could either receive no treatment or treatments unrelated to exercise, with studies comparing different exercise interventions being excluded; iv) outcomes assessed included FBG, 2hPG, HOMA-IR, and HbA1c; v) only randomized controlled trials (RCTs) were considered; vi) there were no language limitations in the included literature.

Exclusion criteria: i) studies such as reports, conference abstracts, and reviews were excluded; ii) excluded from consideration were studies that focused exclusively on aquatic exercise or did not compare land-based exercise to a non-exercise group; iii) studies that administered special drug treatments concurrently with exercise interventions; iv) duplicate experimental data from several publications of the same study were excluded.

**Figure 1:**
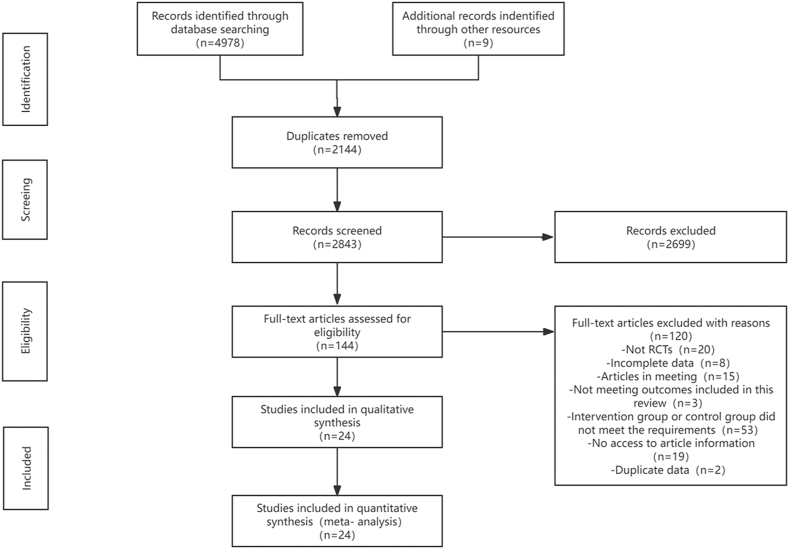
PRISMA study flow diagram.

Two independent reviewers evaluated the titles as well as the abstracts of the literature meeting the inclusion requirement. The study selection process is depicted in Fig. 1.

### Data synthesis and analyses

Two authors independently extracted data. In cases of disagreement, a third author adjudicated, and consensus was achieved through discussion. The inter-rater agreement between reviewers was measured using Cohen’s kappa (κ) coefficient. Data extraction encompassed publication characteristics, methodological features, participant characteristics, exercise details, and outcomes.

Engage Digitizer software was employed to extract data from studies. Data from the last intervention were extracted for studies with multiple follow-up evaluations. Exercise intervention doses in the included trials were evaluated based on ACSM recommendations for cardiorespiratory fitness, muscle strength, and flexibility in apparently healthy adults.

Two authors independently evaluated exercise intervention measures for each study based on ACSM recommendations regarding exercise dose components to assess compliance (see Table 1). Each exercise indicator was scored on a scale of 0–2: 2 points for meeting the standard, 1 point for uncertainty, and 0 points for not meeting the standard. In cases of disagreement, a third author arbitrated to achieve consensus. Using this scoring system, we calculated the proportion of exercise interventions in each study that adhered to ACSM-recommended doses. Studies achieving a compliance ratio of ≥70% were categorised as high compliance, while those below were considered low or uncertain compliance (19, 20, 21).

### Statistical analyses

A meta-analysis using STATA 15.0 compared outcomes across studies, stratifying them into two groups: high compliance and low or uncertain compliance with ACSM recommendations. Due to varied scales in assessing continuous outcomes, the pooled treatment effect size was determined using WMD with a 95% confidence interval (95% CI) under a random-effects model. Statistical significance was set at *P* < 0.05.

Between-study heterogeneity was assessed using the Higgins *I*^2^ statistic and interpreted following Cochrane Handbook guidelines (22): small (*I*^2^ ≤ 25%), moderate (25% < *I*^2^ ≤ 50%), substantial (50% < *I*^2^ ≤ 75%), or considerable (*I*^2^ > 75%). In cases of high heterogeneity, meta-regression analyses were conducted to explore potential sources by examining various research characteristics. Forest plots were used to present results, depicting WMDs for primary and secondary outcomes. Influence analysis assessed the impact of individual study exclusion on meta-analysis results. Publication bias was evaluated using funnel plots, Begg’s rank correlation test, and Egger’s linear regression test. Sensitivity analyses tested the robustness of findings by systematically excluding each study.

### Quality assessment

Independently, two reviewers assessed the methodological quality using the Cochrane Collaboration’s methodology (23), specifically employing the revised Cochrane Risk of Bias tool (Rob 2) (24) for assessing RCTs. The Rob 2 tool comprehensively evaluates risk across several domains: random sequence generation, allocation concealment, blinding of participants and personnel, blinding of outcome assessment, incomplete outcome data, selective reporting, and other potential biases. Bias risk within each domain was categorised as ‘low risk’, ‘some concerns’, or ‘high risk’ (25). Furthermore, we employed the grading of recommendations assessment, development, and evaluation (GRADE) system to gauge confidence in the evidence, classifying it into one of four levels.

## Results

### Study selection

A total of 4,978 articles were initially retrieved from four databases: PubMed (912), Embase (1,434), Web of Science (682), Cochrane (1,950), and other resources (9). After removing duplicates (2,144 articles), 2,843 unique articles remained. Following title and abstract review, 144 articles were selected for full-text reading. Ultimately, 24 articles met the inclusion criteria for this review (Fig. 1). The inter-rater agreement for study inclusion was substantial (*κ* = 0.81).

### Study characteristics

A total of 24 studies, comprising 1,480 participants, included 750 participants in the intervention group and 730 participants in the control group. The age range of the participants was 31 to 63 years. Among these studies, eleven were conducted in China, three in India, three in Finland, and two each in America, Denmark, and Iran, while Egypt had one study. For details, please refer to Table 2.

**Table 2:** Study characteristics.

Author (year)	Country	Sample size (*n*)			Outcomes	ACSM points:	ACSM compliance (%)
		Total/female/male	Age (years) Mean (SD)	Interventions Length of interventions		Cardiorespiratory	
						Resistance	
						Flexibility	
Cai *et al.* (2023) (14)	China	T: 17/9/8	T: 63.41 (5.06)	Yijinjing and RT	FBG, 2hPG	NR	71.4
		C: 17/12/5	C: 61.82 (4.33)	6 months	HOMA-IR	5	
					HbA1c	5	
Chen *et al.* (2021) (13)	China	T: 83/59/24	T: 60.93 (5.71)	AT	FBG, 2hPG	2	33.3
		C: 83/50/33	C: 60.73 (5.83)	24 months	HOMA-IR	NR	
					HbA1c	NR	
Dai *et al.* (2019) (45)	China	T: 37/NR/NR	T: NR	RT and AT	FBG, 2hPG	4	71.4
		C: 35/NR/NR	C: NR	24 months	HbA1c	6	
						NR	
Faerch *et al.* (2021) (46)	Denmark	T: 30/15/15	T: 57.80 (9.90)	Interval training	FBG, HbA1c	4	66.7
		C: 30/17/13	C: 56.70 (8.40)	26 weeks		NR	
						NR	
Hegde *et al.* (2013) (33)	India	T: 14/8/6	T: 46.50 (13.03)	Yoga	FBG, 2hPG	NR	33.3
		C: 15/7/8	C: 44.67 (9.57)	3 months	HbA1c	NR	
						2	
Huang *et al.* (2022) (34)	China	T: 26/20/6	T: 62.00 (5.00)	Yijinjing	FBG, 2hPG	NR	83.3
		C: 21/16/5	C: 63.50 (4.70)	6 months	HbA1c	NR	
						5	
Jadhav *et al.* (2024) (47)	India	T: 79/47/32	T: 47.80 (8.30)	PA	FBG, HbA1c	3	50
		C: 79/52/27	C: 49.90 (6.90)	24 weeks		NR	
						NR	
Liu *et al.* (2013) (30)	China	T: 20/NR/NR	T: NR	Walking and RT	FBG, 2hPG	6	57.1
		C: 21/NR/NR	C: NR	24 weeks	HOMA-IR	2	
						NR	
Luo *et al.* (2023) (11)	China	T: 26 (11:15)	T: 51.00 (9.50)	AT	FBG, 2hPG	4	66.7
		C: 21 (10:11)	C: 50.00 (6.50)	12 weeks	HOMA-IR	NR	
						NR	
Malin & Kirwan (2012) (38)	America	T: 8 (5:3)	T: 45.40 (8.00)	RT and AT	FBG	2	71.4
		C: 8 (6:2)	C: 49.80 (10.90)	12 weeks		8	
						NR	
RezkAllah & Takla (2019) (48)	Egypt	T: 20 (10:10)	T: 31.00 (5.27)	HV-HIIT	FBG, HbA1c	2	33.3
		C: 20 (8:12)	C: 35.90 (5.89)	12 weeks		NR	
						NR	
Ma *et al.* (2022) (12)	China	T: 34 (18:16)	T: 59.18 (3.93)	Baduanjin	FBG, 2hPG	NR	50
		C: 32 (18:14)	C: 59.09 (5.25)	12 months	HbA1c	NR	
						3	
Safarimosavi *et al.* (2021) (49)	Iran	T: 8/NR/NR	T: 38.60 (4.50)	HIIT	FBG, 2hPG	3	50
		C: 8/NR/NR	C: 37.40 (3.20)	12 weeks	HOMA-IR	NR	
					HbA1c	NR	
Venojarvi *et al.* (2013) (35)	Finland	T: 39/NR/NR	T: 55.00 (6.20)	Nordic walking	FBG, 2hPG	4	66.7
		C: 40/NR/NR	C: 54.00 (7.20)	12 weeks	HbA1c	NR	
						NR	
Viskochil *et al.* (2017) (37)	America	T: 9/5/4	T: 46.20 (7.80)	RT and AT	FBG	3	71.4
		C: 8/6/2	C: 49.8 (11.03)	12 weeks		7	
						NR	
Wang *et al.* (2021) (50)	China	T: 82/52/30	T: 59.91 (5.92)	RT	FBG	NR	87.5
		C: 83/50/33	C: 60.73 (5.83)	24 months	HOMA-IR	7	
					HbA1c	NR	
Yan *et al.* (2019) (51)	China	T: 35/20/15	T: 62.06 (8.11)	RT	FBG, 2hPG	NR	87.5
		C: 35/20/15	C: 60.31 (7.56)	12 months	HOMA-IR	7	
					HbA1c	NR	
Zhang *et al.* (2023) (52)	China	T: 25/18/7	T: 61.12 (6.57)	Yijinjing and RT	FBG, 2hPG	NR	71.4
		C: 25/17/8	C: 63.00 (4.72)	6 months	HOMA-IR	5	
					HbA1c	5	
Gidlund *et al.* (2016) (53)	Finland	T: 20/NR/NR	T: 54.00 (6.20)	RT	2hPG	NR	62.5
		C: 17/NR/NR	C: 54.00 (6.90)	12 weeks	HOMA-IR	5	
					HbA1c	NR	
Herzig *et al.* (2014) (54)	Finland	T: 33/24/9	T: 58.10 (9.90)	Supervised walking	FBG, 2hPG	3	50
		C: 35/26/9	C: 59.50 (10.80)	3 months	HOMA-IR	NR	
						NR	
Kurian & Nanjundaiah (2023) (55)	India	T: 12/9/3	T: 37.60 (5.30)	Yoga	FBG	NR	83.3
		C: 12/8/4	C: 40.00 (3.50)	12 weeks	HOMA-IR	NR	
						5	
Skoradal *et al.* (2018) (56)	Denmark	T: 27/NR/NR	IG: 60.00 (6.00)	Football	FBG, 2hPG	2	33.3
		C: 23/NR/NR	CG: 62.00 (6.00)	16 weeks		NR	
						NR	
Liu *et al.* (2021) (57)	China	T: 43/40/3	IG: 60.35 (4.29)	AT	FBG, 2hPG	3	50
		C: 43/39/4	CG: 59.94 (4.40)	12 months	HbA1c	NR	
						NR	
Kargarfard *et al.* (2022) (58)	Iran	T: 22/0/22	IG: NR	AT	FBG, HOMA-IR	4	66.7
		C: 20/0/20	CG: NR	12 weeks	HbA1c	NR	
						NR	

Abbreviation: NR, not reported; T, experimental group, C, control group; FBG, fasting blood glucose; 2hPG, 2‐hour plasma glucose; HOMA-IR, homeostasis model assessment of insulin resistance; HbA1c, glycated haemoglobin A1c; AT, aerobic training; RT, resistance training; PA, physical activity; HIIT, high-intensity interval training; HV-HIIT, high-volume HIIT; IHHT, intermittent hypoxia/hyperoxia training; ACSM, American College of Sports Medicine.

All studies included exercise interventions mainly involving supervised exercise. The types of interventions comprised Yijinjing, Baduanjin, RT, AT, walking, football, yoga, physical activity (PA), interval training, high-intensity interval training (HIIT), and high-volume HIIT (HV-HIIT). The main intervention durations ranged from 3 weeks to 24 months, with exercise frequencies varying from 2 to 5 days per week. After categorising the interventions, nine studies were defined as highly compliant with ACSM recommendations (Table 1). Details of compliance for each individual study are shown in Supplementary Table 1.

### Risk of bias

**Figure 2:**
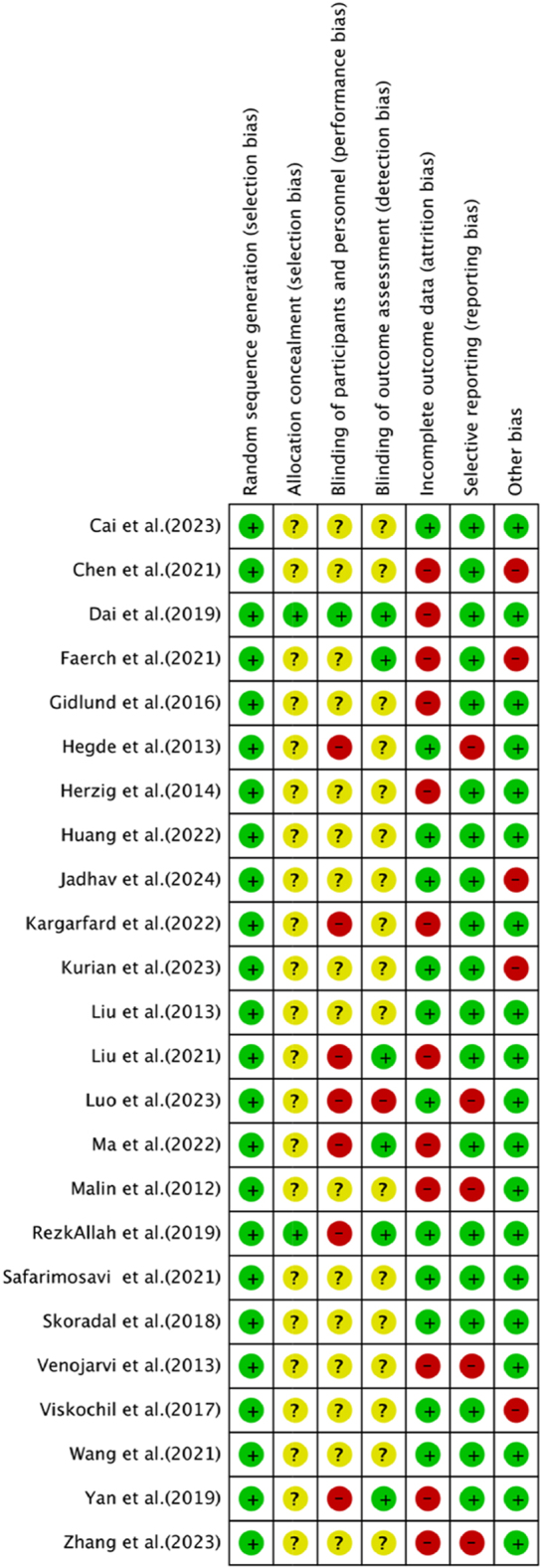
Risk of bias summaries for all exercise trials.

All included studies were assessed as having a low risk of bias for the random sequence generation process. Out of the 24 studies reviewed, only two were deemed to have a low risk of bias regarding allocation concealment. The blinding of participants and personnel presented significant challenges, particularly in exercise-based interventions, making the risk of bias relatively high in this domain. Specifically, seven studies were identified as having a high risk of bias for blinding, only one study was rated as low risk, and the remaining studies fell into the unclear risk category. Regarding the blinding of outcome assessments, six studies utilised randomisation or employed blinded assessors, resulting in a low risk of bias. In terms of incomplete outcome reporting, discrepancies between baseline and post-intervention participant numbers were found in 24 studies; 12 of these studies had no dropouts or minimal dropout rates, hence they were classified as low risk. The risk of selective reporting bias was deemed low in 19 studies. In addition, five studies were identified as having a high risk of other potential biases (Fig. 2). Given that exercise interventions inherently struggle to implement blinding, and considering the significant heterogeneity caused by variations in exercise frequency, duration, and type, we ultimately classified all four indicators as low. For detailed specifications, refer to Supplementary Table 3.

### The impact of compliance with ACSM recommendations on glycaemic control

Among the studies, 9 achieved compliance with ACSM recommendations at or above 70%, while 15 fell below this threshold (Table 1). The lower compliance rates were attributed partly to discrepancies between the exercise interventions and ACSM-prescribed guidelines, and numerous trials lacked detailed descriptions of their treatment protocols. The outcome measure was glycaemic control, which encompassed FBG, 2hPG, HOMA-IR, and HbA1c.

#### FBG

**Figure 3:**
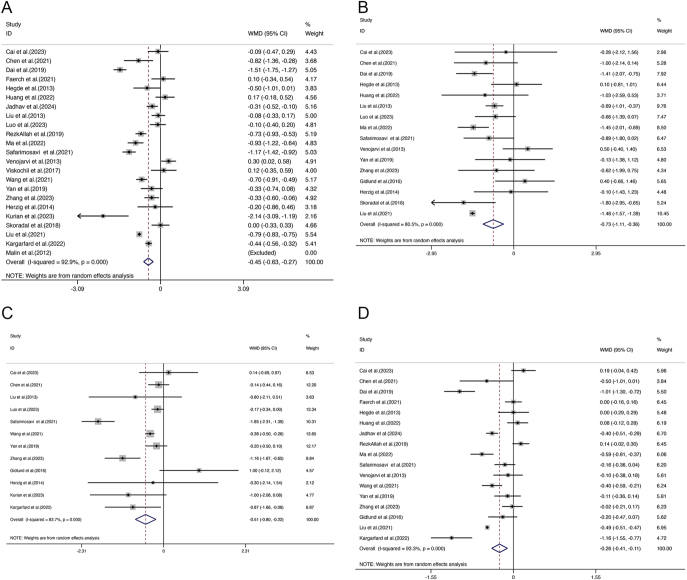
Forest plot of meta-analysis on the effect of exercise on FBG (A), 2hPG (B), HOMA-IR (C), and HbA1c (D) in patients with prediabetic state.

**Figure 4:**
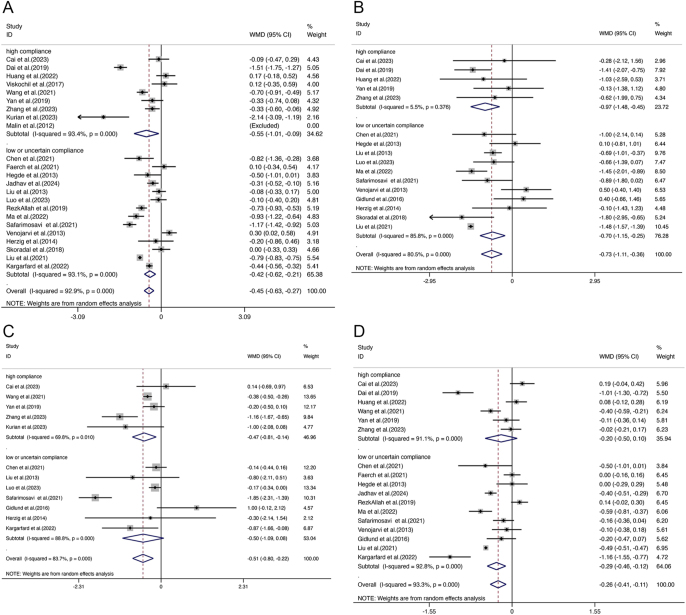
Forest plot of the effect of ACSM compliance on FBG (A), 2hPG (B), HOMA-IR (C), and HbA1c (D) in patients with prediabetic state.

**Figure 5:**
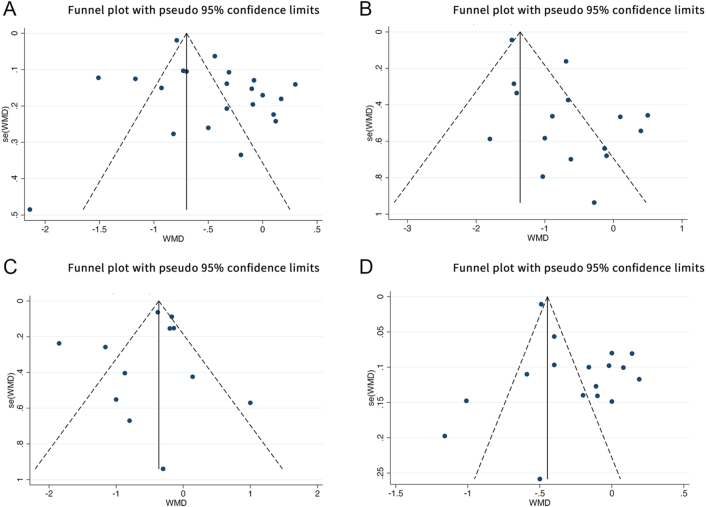
Funnel plot of meta-analysis on the effect of exercise on FBG (A), 2hPG (B), HOMA-IR (C), and HbA1c (D) in patients with prediabetic state.

**Figure 6:**
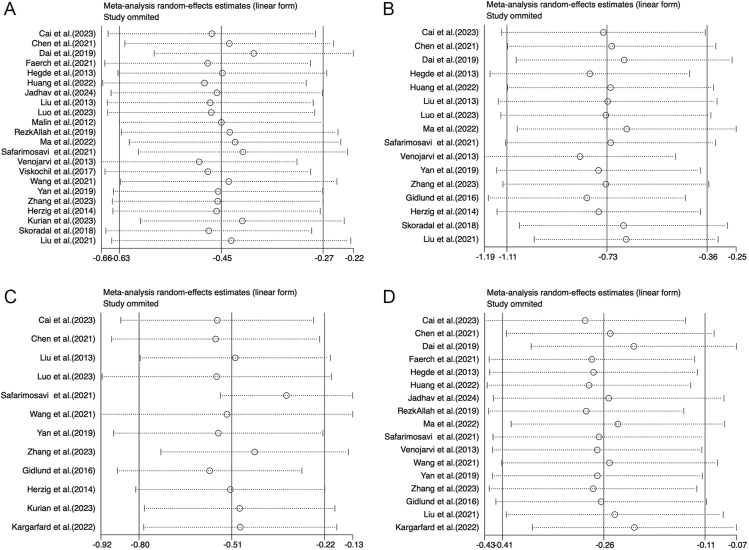
Sensitivity analysis of meta-analysis on the effect of exercise on FBG (A), 2hPG (B), HOMA-IR (C), and HbA1c (D) in patients with prediabetic state.

Analysis of FBG outcomes included 23 studies with a total of 1,443 participants. Of these, 9 studies adhered closely to ACSM guidelines, while the remaining 14 showed low or uncertain adherence. The random-effects model revealed that the overall exercise effect on FBG was −0.45 (95% CI: −0.63, −0.27), with significant heterogeneity at 92.9% (95% CI: 89.5%, 96.3%; *τ*^2^ = 0.1500) (Fig. 3), indicating a meaningful impact of exercise on FBG in the combined sample. Subgroup analysis provided further insights: studies with high adherence to ACSM recommendations showed an FBG effect size of −0.55 (95% CI: −1.01, −0.09) with 93.4% heterogeneity (95% CI: 88.7%, 98.1%; *τ*^2^ = 0.3886) (Fig. 4). Meanwhile, studies with low or uncertain ACSM adherence demonstrated an FBG effect size of −0.42 (95% CI: −0.62, −0.21) with 93.1% heterogeneity (95% CI: 89.3%, 97.0%; *τ*^2^ = 0.1297) (Fig. 4). The Galbraith plot indicates significant heterogeneity among the FBG studies (Supplementary Fig. 1). These results suggest that both high and low adherence to ACSM guidelines positively influence FBG, with confidence intervals excluding zero, indicating statistical significance. Comparing the two compliance levels (FBG: −0.55 vs −0.42) shows that higher compliance with ACSM recommendations generally correlates with a more substantial reduction in FBG. However, the high heterogeneity underscores considerable variability among the studies. Consequently, we performed publication bias assessments and sensitivity analyses. The funnel plot’s visual inspection (Fig. 5) indicated near-symmetry, suggesting no apparent publication bias. Sensitivity analyses, involving the sequential exclusion of individual studies (Fig. 6), confirmed that no single study significantly altered the overall results, supporting the robustness of these findings.

#### 2hPG

For studies focussing on 2hPG, a total of 16 studies comprising 958 participants were analysed. Among these, five studies exhibited high adherence to ACSM guidelines, whereas 11 studies showed low or uncertain adherence. Applying a random-effects model, the aggregate effect of exercise on 2hPG was calculated at −0.73 (95% CI: −1.11, −0.36), with heterogeneity observed at 80.5% (95% CI: 68.0%, 93.0%; *τ*^2^ = 0.3439) (Fig. 3). The Galbraith plot indicates significant heterogeneity among the 2hPG studies (Supplementary Fig. 2). This result suggests that exercise has a significant impact on lowering 2hPG levels in the overall sample. Further subgroup analysis yielded the following: in studies with high adherence to ACSM guidelines, the 2hPG effect size was −0.97 (95% CI: −1.48, −0.45) with a heterogeneity of 5.5% (95% CI: 0%, 64.4%; *τ*^2^ = 0.0224) (Fig. 5). In contrast, studies with low or uncertain adherence showed a 2hPG effect size of −0.70 (95% CI: −1.15, −0.25) with a heterogeneity of 85.8% (95% CI: 75.2%, 96.4%; *τ*^2^ = 0.3973) (Fig. 4). These results indicate that exercise positively impacts 2hPG levels, regardless of adherence level to ACSM guidelines, as the confidence intervals do not include zero, confirming statistical significance. However, higher adherence to ACSM recommendations is generally associated with a more pronounced reduction in 2hPG (−0.97 vs −0.70). The substantial heterogeneity among studies with low or uncertain adherence suggests variability in the results. To address this, we conducted publication bias tests and sensitivity analyses. A visual inspection of the funnel plot (Fig. 5) showed approximate symmetry, indicating no evident publication bias. Sensitivity analyses, involving the exclusion of individual studies sequentially (Fig. 6), demonstrated that no single study significantly influenced the overall results, underscoring the robustness of the findings.

#### HOMA-IR

For studies analysing HOMA-IR, a total of 12 studies encompassing 760 participants were included. Among these, five studies showed high adherence to ACSM guidelines, while the remaining seven exhibited low or uncertain adherence. The random-effects model revealed that the overall impact of exercise on HOMA-IR was −0.51 (95% CI: −0.80, −0.22), with significant heterogeneity at 83.7% (95% CI: 71.4%, 96.0%; *τ*^2^ = 0.1575) (Fig. 3), indicating a notable effect on HOMA-IR across the overall sample. Further subgroup analyses provided these insights: in the high adherence subgroup, HOMA-IR showed a reduction of −0.47 (95% CI: −0.81, −0.14) with 69.8% heterogeneity (95% CI: 39.9%, 100%; *τ*^2^ = 0.0825) (Fig. 4). The Galbraith plot indicates significant heterogeneity among the HOMA-IR studies (Supplementary Fig. 3). Meanwhile, the low or uncertain adherence subgroup demonstrated a HOMA-IR reduction of −0.50 (95% CI: −1.09, 0.08) with 88.8% heterogeneity (95% CI: 79.8%, 100%; *τ*^2^ = 0.4357) (Fig. 4). Regardless of the level of adherence to ACSM guidelines, the confidence intervals of the high adherence subgroup exclude zero, indicating statistical significance. The confidence intervals of the low or uncertain adherence subgroup include zero, confirming statistical insignificance. These findings suggest that higher adherence to ACSM recommendations exercise positively influences HOMA-IR. As the high heterogeneity in both groups suggests considerable variability among the studies, publication bias tests and sensitivity analyses were performed. The funnel plot’s visual inspection (Fig. 5) showed approximate symmetry, indicating no apparent publication bias. Sensitivity analyses, conducted by sequentially excluding individual studies (Fig. 6), revealed that no single study significantly altered the overall results, supporting the robustness of the findings.

#### HbA1c

A total of 17 studies, involving 1,217 participants, were included in the analysis when evaluating HbA1c outcomes. Among these, six studies exhibited high adherence to ACSM guidelines, while eleven studies had low or uncertain adherence. The random-effects model showed that exercise had an overall effect on HbA1c of −0.26 (95% CI: −0.41, −0.11), with a heterogeneity of 93.3% (95% CI: 87.4%, 99.2%; *τ*^2^ = 0.0821) (Fig. 3). This indicates that exercise has a significant impact on reducing HbA1c levels in the overall sample. Subgroup analyses provided further insights: in the high adherence subgroup, the effect size on HbA1c was −0.20 (95% CI: −0.50, 0.10), with a heterogeneity of 91.1% (95% CI: 79.8%, 100%; *τ*^2^ = 0.1270) (Fig. 4). The Galbraith plot indicates significant heterogeneity among the HbA1c studies (Supplementary Fig. 4). Meanwhile, the low or uncertain adherence subgroup showed an effect size of −0.29 (95% CI: −0.46, −0.12), with a heterogeneity of 92.8% (95% CI: 84.8%, 100%; *τ*^2^ = 0.0696) (Fig. 4). Regardless of the level of adherence to ACSM guidelines, the confidence intervals of the low or uncertain adherence subgroup exclude zero, indicating statistical significance. The confidence intervals of the high adherence subgroup include zero, confirming statistical insignificance. These findings suggest that low or uncertain adherence to ACSM recommendations exercise positively influences HbA1c. The substantial heterogeneity observed in both subgroups suggests considerable variability among the studies. Consequently, publication bias tests and sensitivity analyses were conducted. The funnel plot’s visual inspection (Fig. 5) showed approximate symmetry, indicating no significant publication bias. Sensitivity analyses, involving the sequential exclusion of individual studies (Fig. 6), revealed that no single study significantly influenced the overall results, supporting the robustness of the findings.

### Result of meta-regression

Using random-effects meta-regression with REML, we simultaneously evaluated age, baseline HbA1c, and intervention duration as continuous predictors of treatment effects on FBG, 2-h post-prandial glucose (2hPG), HOMA-IR, and HbA1c across included trials. Baseline HbA1c emerged as the most robust effect modifier: each additional 1% unit at entry was associated with progressively larger absolute reductions of 0.52 mmol L^−1^ in FBG (95% CI: 0.29–0.75, *P* < 0.001), 0.68 mmol L^−1^ in 2hPG (0.39–0.97, *P* < 0.001), 0.25 points in HOMA-IR (0.09–0.41, *P* = 0.002) and 0.45% in HbA1c (0.33–0.57, *P* < 0.001), underscoring that interventions confer the greatest benefit among participants with poorer initial glycaemic control. Intervention duration also contributed significantly to outcome heterogeneity: every extra week of treatment yielded incremental decreases of 0.03 mmol L^−1^ in FBG (−0.05 to −0.01, *P* = 0.02), 0.04 mmol L^−1^ in 2hPG (−0.07 to −0.01, *P* = 0.03) and 0.02% in HbA1c (−0.03 to −0.01, *P* = 0.01), whereas the slope for HOMA-IR (−0.01, *P* = 0.08) did not reach conventional significance. Age showed no meaningful influence on any endpoint (all *P* > 0.10), indicating consistent efficacy across the age spectrum studied. Despite adjustment for these three covariates, moderate-to-high residual heterogeneity persisted (residual *I*^2^ 65–72%, *τ*^2^ 0.15–0.58), implying that additional methodological or patient-level factors not captured here continue to drive between-study variance (see Supplementary Table 2 for details).

## Discussion

### Effect of ACSM compliance on the effectiveness of exercise intervention

Our meta-analysis revealed that exercise interventions can enhance glucose metabolism and insulin resistance in individuals with prediabetes. These findings align with established knowledge and previous research (26, 27, 28, 29, 30), affirming exercise’s efficacy as a non-pharmacological treatment for prediabetes. However, some previous research summaries indicate that not all exercise interventions effectively increase glucose metabolism in individuals with prediabetes (31). Factors influencing the effect of exercise on glucose metabolism extend beyond exercise type alone; considerations such as modality, intensity, and dose are also critical. Research indicates that increasing daily average exercise duration is beneficial for enhancing FBG metabolism (32). Whole-body exercises such as yoga, Yijinjing, and Nordic walking have demonstrated positive effects (33, 34, 35).

Unlike previous meta-analyses (16, 36) that overlooked the effects of combined exercise interventions, this study emphasises that incorporating cardiorespiratory, resistance, and flexibility training modalities can offer comprehensive programme recommendations for future studies, moving beyond singular exercise type studies. Nevertheless, there remains a paucity of research on exercise dosage, and uncertainty persists regarding the optimal exercise regimen for treating individuals with prediabetes. According to ACSM recommendations, our study found that high compliance with exercise dosage is more beneficial for improving glucose metabolism and insulin resistance compared with low or uncertain compliance. ACSM-compliant high-compliance exercise interventions encompass various types, such as Yijinjing combined with RT, RT combined with AT, and yoga. Similarly, ACSM-compliant low or uncertain compliance includes AT, RT, interval training, Baduanjin, yoga, PA and HIIT. Our meta-analysis mitigates the impact of predominant exercise types on variations in ACSM compliance. The strength of this study lies in integrating diverse exercise modalities, intensities, durations, and other relevant indicators from previous research. The study employs ACSM compliance as a grouping criterion to assess the impact of exercise dosage on enhancing glucose metabolism and insulin resistance in individuals with prediabetes. This approach provides unique insights into the influence of exercise dosage on prediabetes management, utilising ACSM compliance standards.

Our research aligns with established knowledge and previous research conclusions (33, 37, 38, 39, 40) that exercise serves as an effective non-pharmacological treatment for prediabetes patients. However, we can only infer that exercise improves glucose metabolism and insulin resistance in prediabetes patients, as no specific exercise programme has proven superior to others. A key focus of this study is interpreting ACSM compliance. ACSM-recommended exercise interventions encompass aerobic, resistance, and flexibility exercises, each with detailed recommended exercise dosages. However, descriptions of exercise dosage in RCTs for prediabetes patients are often incomplete or limited to specific types of exercise interventions. For example, out of the nine studies classified as highly compliant with ACSM recommendations, four studies either did not report or inadequately reported the exercise intervention dosage. In addition, among the 15 studies categorised as low or uncertain compliance with ACSM recommendations, eight studies did not report or inadequately reported the exercise intervention dosage. This indicates that a highly compliant exercise intervention dosage according to ACSM recommendations may be incorrectly categorised as low or uncertain compliance. Similar to pharmacological treatments, precise descriptions of exercise prescriptions in interventions are crucial for determining the appropriate range of exercise dosage. While individualised treatment differentiation is necessary during implementation, adjustments should also be made within a reasonable range of exercise prescriptions.

The observation that high-adherence interventions conferred larger reductions in FBG and 2hPG, yet paradoxically smaller HbA1c improvements, aligns with the dose–response framework underpinning exercise physiology and glycaemic control. According to the AMPK–SIRT1 energy-sensor model, each bout of moderate-to-vigorous exercise transiently increases skeletal-muscle glucose uptake via insulin-independent AMP-activated protein-kinase signalling (41). When such bouts are repeated ≥3 times per week at ≥60% VO_2_ reserve – the threshold embedded in the ACSM dose – this acute effect is consolidated into chronic up-regulation of GLUT-4 expression and improved hepatic insulin clearance, manifesting as lower fasting and post-load glucose concentrations (42). The high-compliance subgroup met these thresholds consistently, explaining the 0.13 mmol L^−1^ incremental reduction in FBG compared with low-compliance studies.

Conversely, HbA1c reflects mean glycaemia over ∼8–12 weeks, a window in which inter-study differences in baseline glycaemia, sample size, and measurement error can mask modest between-group differences. The glycation gap hypothesis posits that individuals with similar average glucose may differ in HbA1c by up to 0.5% because of erythrocyte life-span heterogeneity (43). In our meta-analysis, the low-compliance stratum had marginally higher baseline HbA1c (mean 6.1 vs 5.9%), amplifying regression-to-the-mean effects and potentially inflating the apparent benefit of low-dose exercise. This statistical artefact, rather than a true biological superiority, likely explains why low-adherence studies appeared to ‘out-perform’ high-adherence trials for HbA1c while under-performing for acute glycaemic indices.

Finally, behavioural reinforcement theory suggests that participants enrolled in interventions closely aligned with evidence-based guidelines (i.e. ACSM) may exhibit higher self-efficacy and lower drop-out rates, thereby preserving the physiological benefit captured by FBG and 2hPG (44). Collectively, these mechanistic and psychosocial considerations reconcile our seemingly counter-intuitive HbA1c findings and reinforce the importance of adherence to structured, multi-component exercise prescriptions for optimising glycaemic outcomes in prediabetes.

### Limitations of the study and future prospects

First, the study relies on a meta-analysis of existing research, which means it may inherit limitations and biases from the original studies. Since exercise interventions cannot achieve the same level of allocation concealment and double-blind design as medications, most studies were rated as ‘some concerns’ or ‘high’ in the RoB2 domains of ‘allocation process’ and ‘deviation from the protocol’, which may overestimate the effect. Second, while this study confirms that exercise based on ACSM dose recommendations can improve prediabetes-related blood glucose and insulin resistance, the heterogeneity of the original trials limited the derivation of an ‘optimal’ prescription. Accordingly, we recommend adopting a clinically feasible template: ‘moderate-intensity aerobic + RT combined, with 4–5 days per week of ≥150 min aerobic exercise paired with two sessions of full-body RT’. For elderly or obese individuals, the intensity can be reduced and implemented in segmented cumulative sessions. However, we emphasise that data on ethnic minorities, low-income populations, and long-term outcomes remain insufficient. Urgent multi-arm dose–response studies and ≥5-year follow-ups are needed to establish precise, culturally adapted, and sustainable exercise intervention strategies.

## Conclusion

This review underscores exercise as pivotal for improving glucose metabolism and insulin resistance in individuals with prediabetes. Examining optimal exercise doses, we observed that adherence to ACSM recommendations significantly enhanced outcomes in FBG, 2hPG, and HOMA-IR compared with less compliant interventions. Therefore, exercise programme adherence to ACSM recommendations will help individuals with prediabetes persist in or resolve to a normal state. Nonetheless, due to insufficiently detailed exercise protocols in some studies, future research must employ robust experimental designs and larger sample sizes for validation. Notably, low or uncertain compliance subgroups showed superior results in HbA1c.

## Supplementary materials



## Declaration of interest

The authors declare that this work was conducted in the absence of any commercial or financial relationships that could be construed as a potential conflict of interest.

## Funding

This work did not receive any specific grant from any funding agency in the public, commercial, or not-for-profit sector.
